# Two key cathepsins, TgCPB and TgCPL, are targeted by the vinyl sulfone inhibitor K11777 in *in vitro* and *in vivo* models of toxoplasmosis

**DOI:** 10.1371/journal.pone.0193982

**Published:** 2018-03-22

**Authors:** Juan D. Chaparro, Timmy Cheng, Uyen Phuong Tran, Rosa M. Andrade, Sara B. T. Brenner, Grace Hwang, Shara Cohn, Ken Hirata, James H. McKerrow, Sharon L. Reed

**Affiliations:** 1 Department of Pediatrics, Division of Infectious Diseases, Rady Children's Hospital, University of California, San Diego, School of Medicine, La Jolla, California, United States of America; 2 Department of Pathology, University of California, San Diego School of Medicine, La Jolla, California, United States of America; 3 Department of Medicine, Division of Infectious Diseases, University of California, Irvine School of Medicine, Irvine, California, United States of America; 4 Department of Pharmacy, Skaggs School of Pharmacy and Pharmaceutical Science, University of California, San Diego, La Jolla, California, United States of America; University of Sao Paulo, BRAZIL

## Abstract

Although toxoplasmosis is one of the most common parasitic infections worldwide, therapeutic options remain limited. Cathepsins, proteases that play key roles in the pathogenesis of toxoplasmosis and many other protozoan infections, are important potential therapeutic targets. Because both TgCPB and TgCPL play a role in *T*. *gondii* invasion, we evaluated the efficacy of the potent, irreversible vinyl sulfone inhibitor, K11777 (*N-*methyl-piperazine-Phe-homoPhe-vinylsulfone-phenyl). The inhibitor’s toxicity and pharmacokinetic profile have been well-studied because of its *in vitro* and *in vivo* activity against a number of parasites. We found that it inhibited both TgCPB (EC50 = 114 nM) and TgCPL (EC50 = 71 nM) *in vitro*. K11777 also inhibited invasion of human fibroblasts by RH tachyzoites by 71% (p = 0.003) and intracellular replication by >99% (p<0.0001). *In vivo*, a single dose of K11777 led to 100% survival of chicken embryos in an model of acute toxoplasmosis (*p* = 0.015 Cox regression analysis). Therefore, K11777 shows promise as a novel therapeutic agent in the treatment of toxoplasmosis, and may prove to be a broadly effective anti-parasitic agent.

## Introduction

*Toxoplasma gondii* is an intracellular protozoan parasite recognized as a pathogen more than 100 years ago. Humans acquire the parasite mainly through ingestion of contaminated undercooked meat, food or water contaminated with feline feces, vertical transmission from mother to fetus, or through blood transfusions or organ transplants [1]. An estimated 22.5% of the population 12 years and older or 60,000,000 people in the US have been infected with toxoplasmosis, and the seroprevalence is markedly higher in developing countries [2,3]. Although acute infection of an immunocompetent host is usually clinically asymptomatic, it leads to lifelong, latent infection. Furthermore, primary infection of the fetus (approximately 1 in 1000 live births in the US) can cause devastating and even fatal disease [4,5]. Additionally, reactivation of latent infection in immunosuppressed individuals, particularly AIDS patients, can manifest as *Toxoplasma* encephalitis, a uniformly fatal condition if left untreated [2].

The first line of therapy for toxoplasmosis includes a combination of pyrimethamine and sulfadiazine, a regimen more than 50 years old with frequent toxic side effects. Pyrimethamine, a folic acid antagonist, is considered the most effective anti-*Toxoplasma* agent, but it requires monitoring during therapy for bone marrow suppression and is contraindicated during pregnancy due to teratogenicity. Access to pyrimethamine may also be limited by recent significant increases in the retail price [6]. Sulfadiazine, which acts synergistically with pyrimethamine, is a major cause of drug reactions. This is particularly prevalent in the HIV-infected population, where up to 34% of patients receiving prophylactic trimethoprim-sulfamethoxazole experience fever and rash [7]. These percentages rise to as high as 50% in the setting of active AIDS and *Pneumocystis jirovecii* pneumonia [8]. These factors led the CDC to designate toxoplasmosis as one of the neglected parasitic infections in the US in 2014. Thus, drug development to treat toxoplasmosis is an important priority.

*T*. *gondii* tachyzoites can invade any nucleated cell in a process mediated by the sequential release of specialized secretory organelles in an apical complex: micronemes, rhoptries and dense granules. Many of these key proteins require proteolytic processing; more than half of microneme proteins and the majority of rhoptry proteins [9,10] are synthesized as preproproteins that undergo enzymatic maturation before storage and secretion.

Clan CA cathepsins, a subgroup of cysteine proteinases, are potential drug targets as they have been identified as important enzymes in survival of multiple protozoa. *Plasmodium falciparum* utilizes three cathepsin L-like proteinases to digest hemoglobin in the food vacuole [11], and the cathepsin B of *Trypanosoma brucei* is critical for iron acquisition by degrading host transferrin [12]. Unlike most protozoa, *T*. *gondii* only possesses a limited number of Clan CA family C1 cysteine proteinases: one cathepsin B (TgCPB) [13], one cathepsin L (TgCPL) [14], and three cathepsin C’s (TgCPC1, C2 and C3) [15].

Cathepsins are important in invasion of host cells [13, 14, 16–21] and in maintenance of chronic infection by *T*. *gondii* [22]. TgCPL is required for the release of two key microneme proteins, TgM2AP and TgMIC3, which are required for invasion [16], and as a maturase for TgCPB, which is located in the same vacuolar compartment as TgCPL [21]. TgCPB has also been shown to play a role in invasion and replication of the organism in both *in vivo* and *in vitro* models [18]. Both TgCPB and TgCPL were upregulated in mice with chronic toxoplasmosis infection [23].

The vinyl sulfone inhibitor, K11777 (N-methyl-piperazine-phenylalanyl-homophenylalanyl-vinylsulfone phenyl), has activity against both protozoan cathepsin Bs (*Trypanosoma brucei)* [12], cathepsin Ls (*Trypanosoma cruzii)* [24], and cathepsins which are structurally cathepsin L, but have substrate specificities of cathepin Bs (*E*. *histolytica*) [25]. K11777 is a well-tolerated, orally bioavailable compound that is currently in the late stages of pre-clinical development for treatment of infection with *Trypanosoma cruzi* (McKerrow, personal communication). We now show that K11777 significantly blocks invasion and multiplication of *T*. *gondii in vitro*, and that a single dose dramatically prevents mortality in a chick model of toxoplasmosis.

## Materials and methods

### *Toxoplasma gondii* cultures

Primary human foreskin fibroblasts (HFF, HFF-1 SCRC-104I from ATCC, Manassas, VA) were cultured in Dulbecco’s Modified Eagle’s Medium (DMEM, Cellgro) containing 10% heat-inactivated fetal bovine serum (Hyclone, Thermo Fisher Scientific, Waltham, MA) and penicillin and streptomycin (50μg/mL). *Toxoplasma gondii* RH (a kind gift from Dr. John Boothroyd) was maintained by serial passage in human foreskin fibroblasts (HFF) monolayers as previously described [15].

### Reagents

All reagents were obtained from Sigma (St. Louis, Mo.) unless otherwise noted. K11777 was made in its HCl salt form by Seres Laboratories, Inc. (Santa Rosa, CA), and stock solutions made at 20 mM or 100 mM in DMSO.

### *In vitro* invasion and replication assays

Human foreskin fibroblasts (HFF) were grown to near confluency in 8-well chamber slides in preparation for the invasion and replication assays (Labtek, Scotts Valley, CA). To assess the effect of K11777 on the ability of tachyzoites to invade, RH tachyzoites (5 X 10^5^) were added to each well of confluent chamber slides in either media alone or with 20μM K11777, and allowed to invade for 2 hours. The wells were fixed, stained with acridine orange, and the number of invaded fibroblasts determined in a minimum of 100 cells as previously described [13]. For inhibition of replication, 2x10^5^ RH tachyzoites were first allowed to invade each well of confluent chamber slides in complete media for two hours, the media was removed, and the slides were washed thoroughly with PBS to remove free tachyzoites. The wells were then replenished with either complete media alone or with complete media and K11777 at 20μM and allowed to incubate for 24 hours total. The slides were fixed, stained with acridine orange, and the number of tachyzoites/vacuole were counted as previously described [13].

A comparison was performed of detection of parasite invasion by acridine orange staining with a modification of the red/green invasion assay [16, 26]. Extracellular parasites were stained with p30 mAb to the surface antigen, SAG1 [27], (Abcam, Cambridge, MA, 1:10 dilution), then permeabilized and incubated with rabbit anti- ROP13 Ab (1:2000 dilution, kind gift of Dr. Peter Bradley). Secondary antibodies included goat, anti-mouse AlexaFluor 594 (1:2000 dilution) and goat, anti-rabbit AlexaFluor 488 (1:500 dilution, Abcam, Cambridge, MA).

### Effect of K1777 on human foreskin fibroblasts (HFF)

We generated confluent monolayers of HFF host cells in clear bottom 96-well plates. These cells were treated with 10-fold dilutions of K1177 (2 mM–0.02 μM). After 1 or 7 days incubation, the resulting cytotoxicity was measured with the Cell Titer 96 Non-Radioactive Cell Proliferation Assay according to manufacturer’s instructions (Promega, Madison, WI). The cell viability values were calculated relative to the untreated controls (defined as 100% survival) using a multi-mode plate reader (SpectraMax i3X, Molecular Devices, SoftMax Pro 7.02) [28]. The EC50 was calculated from the dose-response curve in GraphPad Prism.

### Production and inhibition of active recombinant *T*. *gondii* cathepsin B and L

Recombinant TgCPB and TgCPL proteins were expressed in *Pichia pastoris* under the control of the *Pichia* methanol-inducible, alcohol oxidase (AOX1) promoter and the yeast α-factor secretion signal (Invitrogen) as previously described (S1 Table) [14]. Inhibition of recombinant TgCPB and TgCPL by K11777 was tested by incubating the recombinant proteinase with varying dilutions of K11777 at room temperature for 10 minutes and then measuring the cleavage and liberation of the fluorescent leaving group AMC from synthetic peptide substrates as described above. A dose-response curve was then created in GraphPad Prism to determine the EC50 as previously described [28].

### *In vivo* model of K11777 activity against acute toxoplasmosis

Fourteen-day old pathogen-free fertilized chicken eggs (McIntyre Farms, Lakeside, CA) were inoculated with 10^4^ RH tachyzoites suspended in either 50μL of cell culture medium alone or in medium with K11777 to achieve a concentration of 20 μM in the estimated blood volume of a 14-day old embryo [29]. The tachyzoites were injected directly into the chorioallantoic vein using a 28-gauge needle as previously described [18]. Infection was allowed to proceed until death or day 7 post-inoculation (before hatching). Livers and brains were harvested from all embryos at 7 days post-infection or as soon as death occurred, as evidenced by lack of movement or decreased prominence of vasculature. One half of each organ was fixed in 4% paraformaldehyde for histopathology (hematoxylin/eosin staining and immunostaining for *T*. *gondii* with anti-*T*. *gondii* HRP antibody). The remaining half of each organ was used for real-time PCR.

### Ethics statement

As per Public Health Services policy, Institutional Animal Care and Use Committee oversight is not required for the chick embryo model of toxoplasmosis using unhatched eggs. Although avian species develop vertebrae in their development prior to hatching, the Office for Laboratory Animal Welfare has interpreted “live vertebrate animal” to apply to avians (e.g., chick embryos) only after hatching (approximately 21 days). The UCSD Institutional Animal Care and Use Committee agrees with these guidelines. Available from: http://grants.nih.gov/grants/olaw/references/ilar91.htm

### Quantification of *T*. *gondii* in chick model of acute toxoplasmosis

The single copy SAG1 gene [27] (GenBank accession no. X14080) was used as the reference gene to calculate parasite load in tissue samples relative to a standard curve [18]. The standard curve samples were obtained by adding 10^7^ RH tachyzoites to 100 mg samples of uninfected brain or liver from 19-day old chick embryos (unhatched) and homogenizing the preparation with a cordless homogenizer (VWR) in kit lysis buffer. Total genomic DNA was extracted from 25 μL aliquots using the DNeasy Kit according to the manufacturer’s instructions (Qiagen, Germantown, MD). Extracted genomic DNA was eluted in 200 μL of elution buffer and serially diluted (10^3^−10^5^), and the threshold cycle values determined for the standard curve. The plot of the serial dilutions vs. the threshold cycle was generated for each experiment by the StepOnePlus Real-Time PCR System (Applied Biosystems, Foster City, CA) and had an average correlation coefficient (R^2^) of 0.993 and efficiency of 84.53%. Experimental tissue samples were prepared by weighing out 100 mg of tissue (liver or brain), which was homogenized and genomic DNA extracted as above. Two microliter aliquots of genomic DNA were used as template in technical triplicates with primers and probe sets (S1 Table) designed using the Integrated DNA Technologies website (https://www.idtdna.com/site) and MESA GREEN qPCR MasterMix Plus (AnaSpec Inc., Fremont, CA) on the StepOnePlus Real-Time PCR System (Applied Biosystems, Foster City, CA). Cycling conditions were 95°C for 10 min, followed by 40 cycles of 95°C for 15s, and 60°C for 1 min.

### Statistical analysis

Statistical analysis was performed using GraphPad Prism software 6.0 and IBM SPSS Statistics Version 21. Two-tailed independent samples T-tests were used to compare continuous variables (p<0.05), Chi-square analysis was utilized for comparing ordinal groups and survival analysis was performed using Cox regression analysis.

## Results

### Efficacy of K11777 against recombinant TgCPL and TgCPB

Recombinant TgCPL and TgCPB proteins were activated with DTT, and their baseline protease activity was compared to activity when logarithmic dilutions of K11777 were added to create dose-response curves. The resultant EC50 value of K11777 against TgCPL was 71nM and against TgCPB was 114nM.

### K11777 inhibits invasion and intracellular multiplication

**Fig 1:**
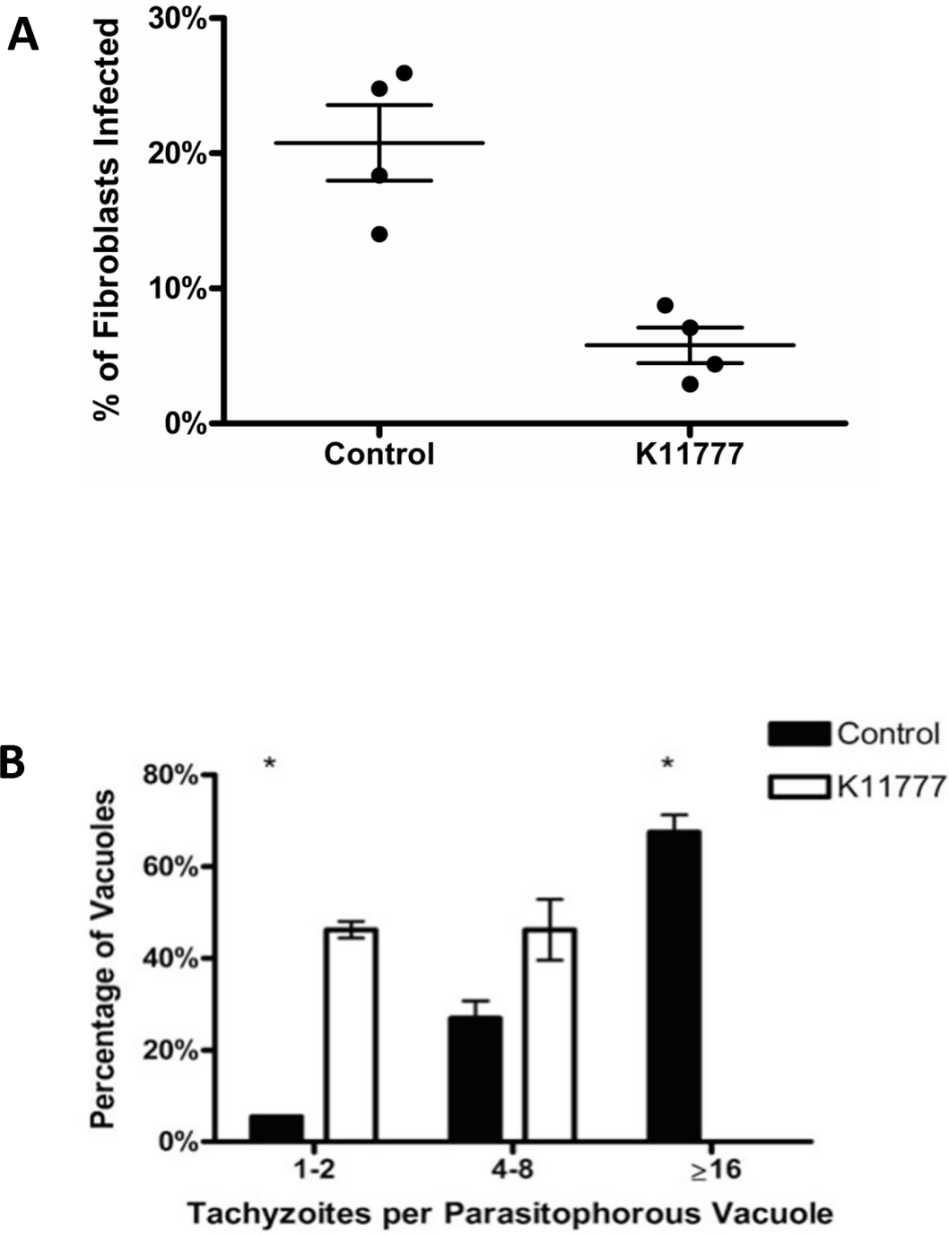
(A) Effect of K11777 on tachyzoite invasion. The percentage of fibroblast cells invaded by RH strain tachyzoites after 2 hrs in complete media alone (Control) was compared to invasion in the presence of K11777 (20μM in complete media). K1777 significantly inhibited invasion of the fibroblasts (N = 8 for each treatment, p = 0.003). Error bars reflect SEM. (B) Effect of K11777 on intracellular tachyzoite replication. RH strain tachyzoites (2 X 10^5^) were allowed to invade HFF in chamber wells for 2 hrs in media alone, the free tachyzoites washed away, and either media alone (control) or media with K11777 (20 μM) was added for an additional 22 hrs. When the number of tachyzoites per parasitophorous vacuole was determined, the K11777 treated wells had significantly more parasitophorous vacuoles with only 1–2 tachyzoites and none with ≥ 16/ vacuole. (N = 8 for each treatment.) * Significant at p<0.001 by Chi-square. Error bars reflect SEM.

The addition of K11777 to cell culture medium at a concentration of 20μM led to a significant decrease in invasion during the two-hour invasion period (Fig 1A). The control wells had an average of 20.8% of fibroblasts infected, and the wells with K11777 had 5.8% of fibroblasts infected, representing a relative reduction of 71% (p = 0.003). Equivalent results were found whether invasion assays were performed and analyzed by acridine orange staining or differential staining of internal and external tachyzoites in the red/green invasion assay [16, 26] (S1 Fig).

The rate of intracellular replication was also significantly inhibited by K11777 with a relative reduction of >99% of the number of cells with ≥16 tachyzoites/ vacuole in K11777 treated cells vs. control RH (p<0.0001) (Fig 1B). There was no significant difference in the number of cells containing 4–8 tachyzoites (p = 0.272).

To confirm that K11777 did not affect the fibroblast cells, we measured the cytotoxicity of K11777 for confluent monolayers of HFF host cells in clear bottom 96-well plates incubated for 24 hrs with 10-fold dilutions of K1177 (2 mM–0.02 μM). Cytotoxicity was measured by formazan uptake using the CellTiter96 Non-radioactive Cell Proliferation Assay (Promega, Madison WI) and compared to % survival of untreated controls [28]. The EC50 was 94 μM at 24 hrs or 4.7-fold higher than the concentration used in the experiments.

### K11777 protects chick embryos from fatal *Toxoplasma* infection

**Fig 2:**
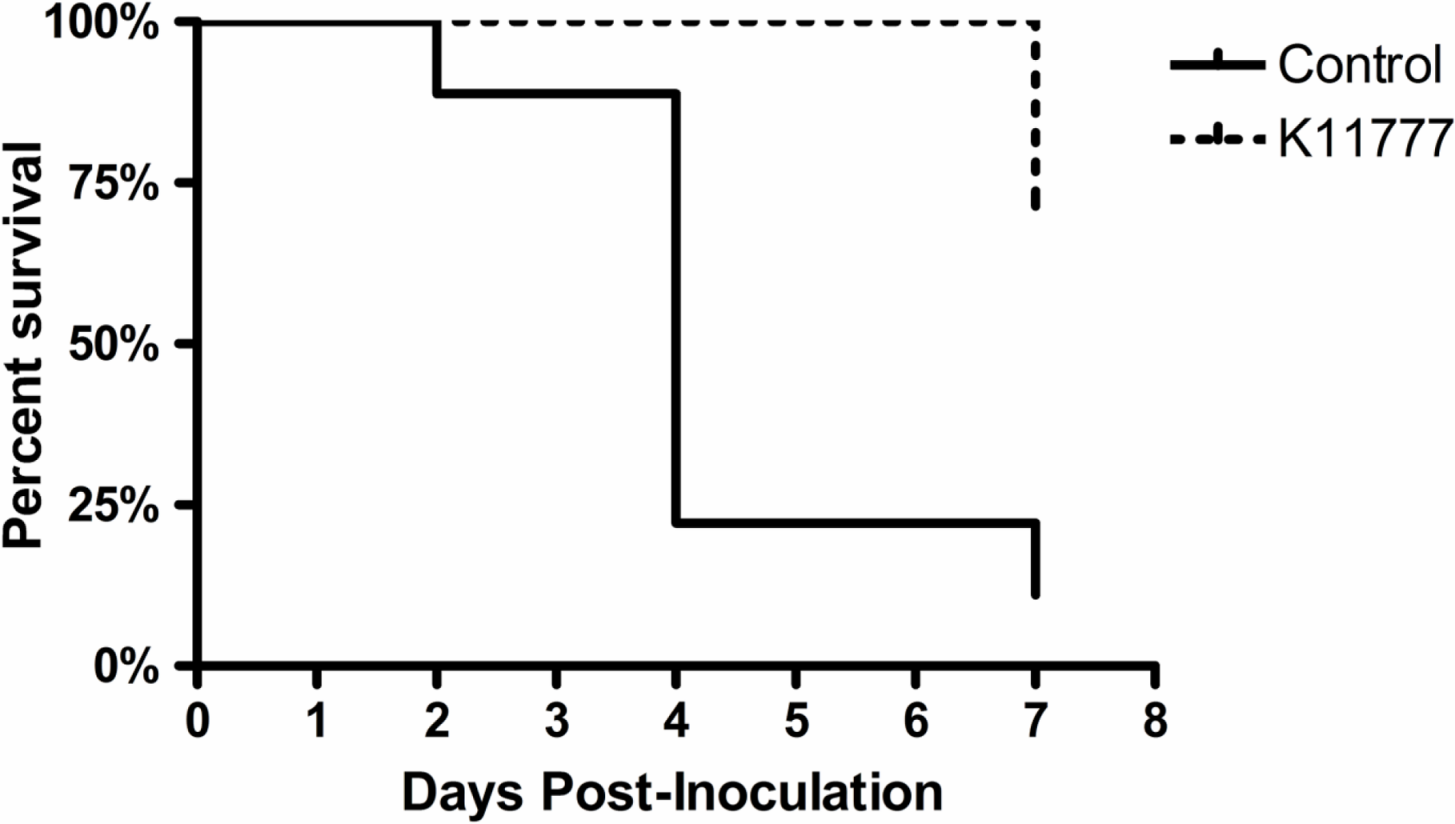
A single dose of K11777 protects chicken embryos in an *in vivo* model of toxoplasmosis. Fourteen day old chick eggs (N = 10/ treatment) were injected through the chorioallantoic vein with 10^4^ RH tachyzoites in media alone (Control) or media containing K11777 for a final blood concentration of 20 uM). The Kaplan-Meier Survival curve of the chick embryos (N = 10 per group) showed that 100% of the chick embryos treated with a single dose of K11777 embryos survived until Day 6 vs. only 20% of the control embryos (p = 0.015 by Cox regression analysis).

Chick embryos were injected with 10^4^ tachyzoites in growth media alone or with a single dose of K11777 for a final blood concentration of 20 μM in the chick. Eggs were allowed to proceed to day 21 or until death. The survival curve of the two groups is displayed in Fig 2. All of the treated chick embryos survived until day 6 *vs*. only 2 of the controls (p = 0.015 by Cox regression analysis).

**Fig 3:**
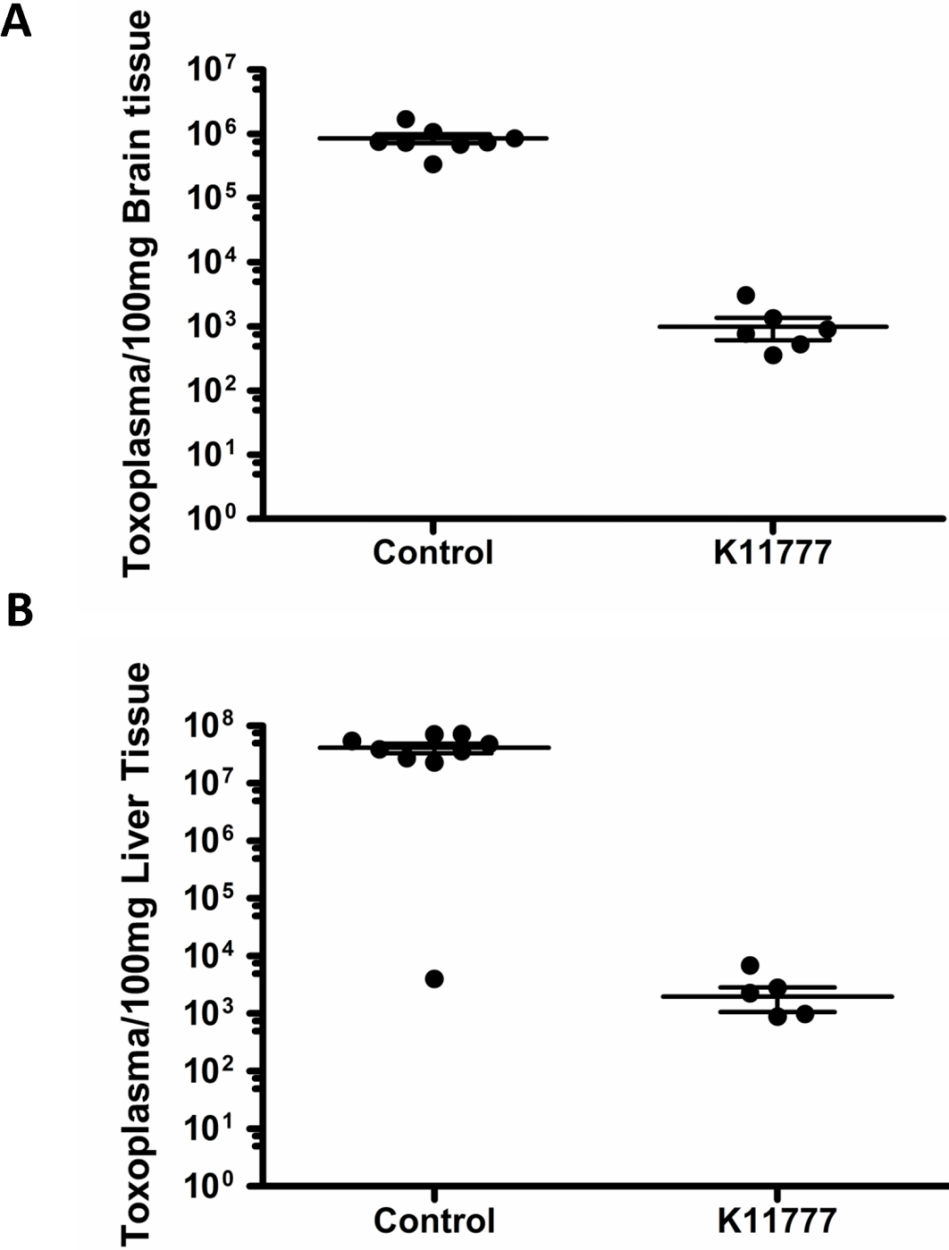
(A) A single dose of K11777 reduces parasite load in the brain of chicken embryos. K11777 treated chicken embryos (N = 10) (final blood concentration 20 μM) showed a 3-log reduction in parasite burden in brain tissue as measured by quantitative PCR compared to chicken embryos injected with parasites in media alone (N = 10) (Control). Error bars reflect SEM. (B) A single dose of K11777 reduces parasite load in the liver of chick embryos. K11777 treated chicken embryos (final blood concentration 20 μM) showed a 4-log reduction in parasite burden in liver tissue as measured by quantitative PCR compared to chicken embryos injected with parasites in media alone (Control). Error bars reflect SEM.

Quantitative PCR of liver and brain tissue harvested from the chick embryos revealed a 3–4 log drop in parasite load in the treated embryos. The average number of *Toxoplasma* in brain tissue (100 mg) was 8.53x10^5^ in controls vs. 9.92x10^2^ in treated (p <0.001, Fig 3A) and in liver (100 mg), 4.12x10^7^ in controls vs. 1.98x10^3^ in treated (p = 0.001, Fig 3B). These differences were highly significant and reflect a ≥ 99.9% reduction in parasite burden in the K11777 treated group.

## Discussion

*T*. *gondii’s* very limited repertoire of only five papain-like cathepsins, one cathepsin B, one cathepsin L, and three cathepsin Cs, is unique among parasites [15]. The role of TgCPL (CPL) in *T*. *gondii* biology and host-parasite interactions has been the best characterized. Carruther’s group has shown that TgCPL is required for release of key microneme attachment proteins [16] and plays a role in the maturation of TgCPB [19]. Knock-outs of TgCPL in a cyst producing strain, ME49, revealed a role of TgCPL in autophagy required for the maintenance of chronic infection [22].

The physiologic substrate(s) of TgCPB have not been definitively identified. We had earlier reported that TgCPB localized to the rhoptries and specific inhibitors of TgCPB decreased degradation of ROP2, a major rhoptry protein that is proteolytically processed [13]. This work predated the characterization of TgCPL, and with newer antibodies and organelle markers, Carruther’s group reported that TgCPB co-localized with TgCPL in a new vacuole (VAC), which is proximal but not part of the rhoptries [19]. Both TgCPB and TgCPL are upregulated during chronic mouse infection [23], but knock-outs of the TgCPL or TgCPB genes are not lethal [21]. Thus, TgCPB may add cathepsin redundancy that is present in all higher eukaryotes.

Because both TgCPB and TgCPL play a role in invasion [13, 18–21], we looked for inhibitors of both cathepsins. We focused on the vinyl sulfone derived compound, K11777, because it has activity against both B and L type cathepsins in other parasites [24–25, 30–37] and has undergone pharmacology and toxicity testing in cell culture, mice, rats, dogs and non-human primates [30]. We found that K11777 was active against both active recombinant TgCPL (EC50 = 71nM) and TgCPB (EC50 = 114nM. *In vitro* experiments showed that K11777 (20 μM) significantly blocks both invasion and intracellular multiplication (Fig 1). Most impressively, a single dose of K11777 (final blood concentration of 20 μM) at the time of infection with tachyzoites prevented mortality in a chick embryo model of acute infection (Fig 2), resulting in a multi-log reduction in total parasite load (live and dead parasites) in the liver and brain (Fig 3).

K11777 has undergone extensive toxicity and pharmacokinetic studies because of its promise as a new drug for Chagas’ Disease [24, 30–31]. Although we used K11777 intravenously, the drug is orally bioavailable in rats (37%) and dogs (30%) [30]. Oral doses of 200 mg/kg in cynomolgus monkeys had a half-life of 5 hours and caused some elevation of liver function tests [30]. Subsequent studies in rats determined that elevation of liver function tests occurred only at doses greater than 150 mg/kg and were reversible. The estimated dose for humans is only 4 mg/kg, and the drug is in late stage preclinical studies for treatment of Chagas’ Disease (JH McKerrow, personal communication). CNS penetration by K11777 has not been determined, but based on its structure, is unlikely. Optimization of the scaffold is ongoing, and further studies in mouse models with chronic infection must still be performed.

K11777 also shows promise as a broad-spectrum anti-parasitic agent. It was first identified during screening of cathepsin peptide inhibitors against, cruzain, a key cathepsin L of *T*. *cruzi* [30–31]. K11777 is also effective *in vitro* and *in vivo* in a number of parasites which all have a central cathepsin B or L linked to pathogenicity including schistosomiasis [32], amebiasis [33], *Tritrichomonas foetus* [34], *Leishmania tropica* [35], hookworm [36], and *Cryptosporidium parvum* [37]. Our current findings demonstrate that TgCPB and TgCPL are valid drug targets in *Toxoplasma* and provide further support for the importance of developing K11777 as a novel, antiparasitic drug.

## Supporting information

S1 Fig Determination of *T*. *gondii* invasion with acridine orange (AO) staining compared to the red/ green invasion assay (RG). Rh tachyzoites (2X 10^5^) were allowed to invade HFF in chamber slides for 2 hrs in complete medium (Control) or in the presence of K11777 (20 μM). Invasion was compared by acridine orange staining or with mouse p30 Ab for external tachyzoites detected with Alexa 494 (red) or internal tachyzoites after permeabilization with rabbit anti-ROP 13 AB detected with Alexa 488 (green). Comparable invasion rates were observed with acridine orange staining and the red/green assay (N = 4 per condition, p>0.05).
(TIF)

S1 Table List of primers used in experiments. Refer to manuscript for details.
(DOCX)
