# Supplementary figures and images for: Two key cathepsins, TgCPB and TgCPL, are targeted by the vinyl sulfone inhibitor K11777 in in vitro and in vivo models of toxoplasmosis
Source: PLoS One. 2018 Mar 22;13(3):e0193982. doi: 10.1371/journal.pone.0193982 (PMC5863946; doi:10.1371/journal.pone.0193982)

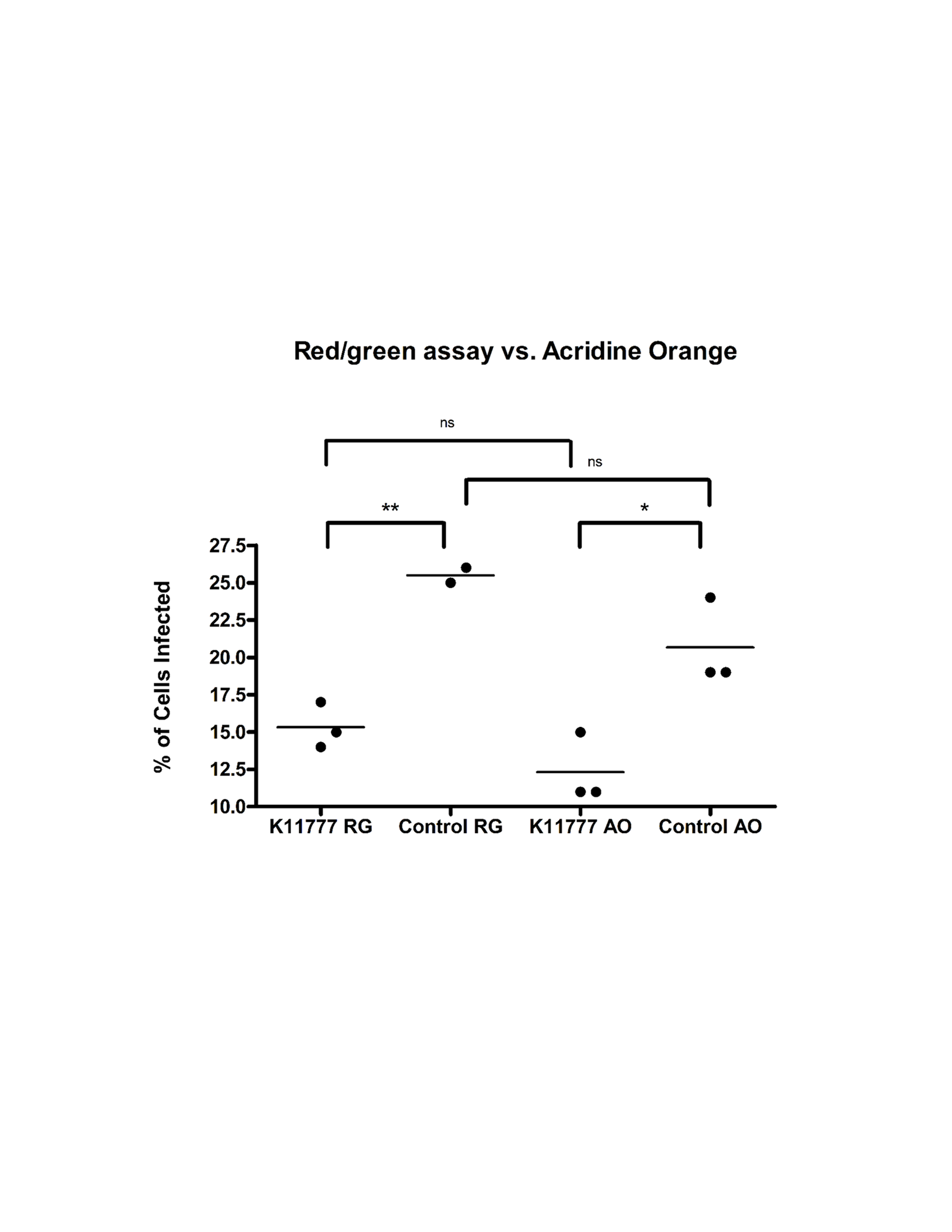

Supplement: S1 Fig — Rh tachyzoites (2X 105) were allowed to invade HFF in chamber slides for 2 hrs in complete medium (Control) or in the presence of K11777 (20 μM). Invasion was compared by acridine orange staining or with mouse p30 Ab for external tachyzoites detected with Alexa 494 (red) or internal tachyzoites after permeabilization with rabbit anti-ROP 13 AB detected with Alexa 488 (green). Comparable invasion rates were observed with acridine orange staining and the red/green assay (N = 4 per condition, p>0.05). (TIF) [file pone.0193982.s001.tif]
