# Supplementary material for: Two key cathepsins, TgCPB and TgCPL, are targeted by the vinyl sulfone inhibitor K11777 in in vitro and in vivo models of toxoplasmosis
Source: PLoS One. 2018 Mar 22;13(3):e0193982. doi: 10.1371/journal.pone.0193982 (PMC5863946; doi:10.1371/journal.pone.0193982)
Supplement: S1 Table — Refer to manuscript for details. (DOCX) [file pone.0193982.s002.docx]

| Primer | Function | Target | Sequence |
| --- | --- | --- | --- |
| 1 | recombinant pichia rTgCPB | rTgCPB forward | 5′-CTCGAGAAAAGAACCCCGGACGACTCGTTGTTTCCGCTT-3’ |
| 2 | recombinant pichia rTgCPB | rTgCPB reverse | 3′-GCGGCCGCCTACATTTCTCTCTCCTCTTCTGC-5’ |
| 3 | recombinant pichia rTgCPL | *rTgCPL forward* | 5’-GAATTCATGACAGCAGCGAGACGCACTAC-3’ |
| 4 | recombinant pichia rTgCPL | *rTgCPL reverse* | 5’-GCGGCCGCTCACATCACGGGGAAAGACGCATCT-3’ |
| 5 | tachyzoite quantification primers | SAG1 F | 5′-GTCATTGTAGTGGGTCCTTCC-3′ |
| 6 | tachyzoite quantification primers | SAG1 R | 5′-GCCTCATCGGTCGTCAATAA-3′ |
| 7 | tachyzoite quantification primers | SAG1 PrimeTime Probe | 5’-TCCTACGGTGCAAACAGCACTCTT-3’ |
